# Mapping disability and climate change knowledge base in Scopus using bibliometric analysis

**DOI:** 10.4102/ajod.v13i0.1339

**Published:** 2024-03-22

**Authors:** Tawanda Makuyana, Kaitano Dube

**Affiliations:** 1 Department of Tourism and Integrated Communication, Faculty of Human Sciences, Vaal University of Technology, Vanderbijlpark, South Africa

**Keywords:** disability, climate change, persons with disabilities, inclusive climate change framework, extreme weather events

## Abstract

**Background:**

Climate change and disability are rarely addressed by academic scholars within the spectrum of disabilities and as a single field of study. However, the intersectionality of disability exacerbates the vulnerability of people with disabilities to climate change as climate change frameworks in the Global North and South continue excluding them.

**Objectives:**

This study aims to map the research-based knowledge housed in Scopus on disability and climate change. At the same time, it provides insights into innovative (novelty) ways of thinking and proposes a futuristic research agenda.

**Method:**

A bibliometric analysis was conducted on Scopus-indexed articles using VOSviewer to map co-occurrences of keywords and co-authorship, and a manual thematic-scoping review augmented the data analysis.

**Results:**

The disability and climate change debate as a joint study evolved from concern among health practitioners to human rights and social inclusion.

**Conclusion:**

In conclusion, there is a skewness towards mental health and medical sociology lens, while other sub-groups of persons with disabilities are yet to be engaged in co-creating disability-inclusive climate change knowledge.

**Contribution:**

Thematic areas emerged as gaps that future studies embed principles enshrined in the United Nations Convention for the Rights of Persons with Disabilities and the Sustainable Development Goals.

## Introduction

The disability movement and scholarly debate on disability emerged in the Global North in the 1900s (Ben-Moshe 2020; Brune 2019). By then, the narrative seeks inclusion and alleviation of ‘othering’ attitudes and societal practices using phrases like the liberation of the person with disabilities (Ben-Moshe 2020). The medical and medical sociology perspectives on disability are prevalent among organisations like hospitals, rehabilitation, and care centres (Meyers, 2022). With time, disability evolved into human rights advocacy using legislative tools like anti-discriminatory disability laws like the *Americans with Disabilities Act of 1990* in the United States of America. Later, the United Nations Convention for the Rights of Persons with Disabilities of 2006 (UNCRPD) (United Nations, 2006) combined all perspectives, including the social lens (Guffey & Williamson 2020). However, this never claimed to be a panacea to the vulnerability of persons with disabilities, but a complementary initiative towards providing principles and/or guidelines for societal cohesion and the need to contextualise disability in communities, environmental settings, socio-economic frameworks and research.

While states are translating UNCRPD into domestic legislative frameworks (Nzo et al., 2023), disability and climate change are still not commonly found in single-joint debate, even on built-environments practices and academic fraternity. This results in a scarcity of scientific literature that informs policy and practices that embed disability-inclusive climate change resilience (Bell, Tabe & Bell 2020). On the one hand, organisational-based projects and reports like those by Fong (2022) reflect disability and climate change as separate discussions. It implies that even in organisational reports, climate change and disability are rarely embedded in transformative approaches. Therefore, in single studies, socio-economic inclusion within the organisation-based frameworks that respond to global warming, extreme weather events and health hardly includes or involves persons with disabilities (Kett et al., 2021). On the other hand, organisations for persons with disabilities (OPDs) are advocating for inclusive disaster-management frameworks (Shakespeare et al., 2021) and the impacts of climate change (Atwoli, Muhia & Merali 2022). However, these realities implicate and worsen the vulnerability of persons with visible and hidden disabilities.

The Intergovernmental Panel on Climate Change (IPCC 2021) explains climate change as a process that influences the transformation of the climate observed by varying mean and variability of weather patterns over a long period, typically decades or longer. It implies that unless disability and climate change foster joint narratives, compounded vulnerability among persons with disabilities can be worse because of climate change-induced socio-economic costs (Frame et al. 2020).

‘Persons with disabilities include those with long-term physical, mental, intellectual or sensory impairments which in interaction with various barriers may hinder their full and effective participation in society on an equal basis with others’ (United Nations, 2006). Therefore, disability is the complex interaction between the ways that disability is constructed (individual and social constructivism), experienced, the interplay between individual interpretation and shared understanding, the private versus social self, and the politics of impairment versus socio-environmental disablement (Olsen & Pilson, 2022).

The study is motivated to assess how scientific research has delved into disability and climate change in response to interrelated Sustainable Development Goals (SDGs) and the UNCRPD. The UNCRPD and SDGs came into existence to guide the global community challenges for research-based knowledge to inform and facilitate alleviating the impacts through policy and practice. Hence, the present study can contribute to the research agenda to augment existing geospatial depth and dimensions covered in disability and climate change knowledge co-creation. Ultimately, it provides insights into equitable coverage of disability inclusion within climate change conversations.

Understanding the available coverage of disability and climate change as a joint field of study (progress and gaps) contributes to shedding light on multi-dimensions, layers, trends, concerns and research constituents (Donthu et al. 2021). It, therefore, furthers the debate on and for novelty knowledge, practice and research-based initiatives on disability and climate change as a joint social field (Bell et al. 2020; Dietz, Shwom & Whitley 2020). It implies that the present bibliometric study aims to be a science mapping research using co-word analysis and co-authorship analysis on Disability and climate change as a joint field of study. VOSviewer-visualisation presented the data from the international research platforms indexed by the Scopus database because it can handle extensive scientific data (Donthu et al. 2021). Therefore, a bibliometric analysis using Scopus enables the study to uncover a one-stop overview while identifying knowledge gaps and research dimensions to propose a research agenda on disability and climate change themes (Donthu et al. 2021).

## Research methods and design

### Approach

**FIGURE 1:**
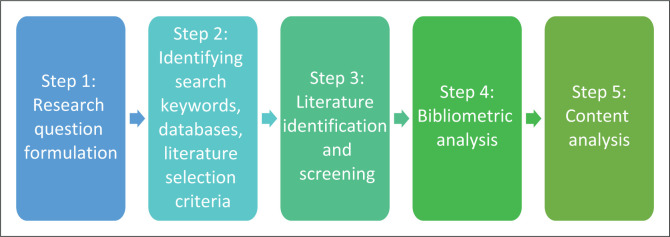
Five-step process used for this study.

The bibliometric analysis was a triangulated systematic review. It, therefore, followed Shen and Lai’s (2022) view that a systematic quantitative approach fosters better objective coverage of disability and climate change as a joint field of study compared to traditional pure qualitative approaches. According to Pickering and Byrne (2014), the systematic quantitative review enables mapping the knowledge boundaries and identifying research gaps in a research area. In this instance, the systematic quantitative review adopted a five-step approach proposed by Mascarenhas et al. (2018) and Pickering and Byrne (2014), as illustrated in Figure 1.

Figure 1 illustrates the steps taken in the present study, as further explained in the subsequent sections.

### Bibliometric procedure

The study implements the steps in identifying and screening data from Scopus, as illustrated in Figure 1. The authors defined the aim of science mapping research on co-word analysis and co-authorship analysis on disability and climate change as a joint field of study. The study would uncover themes in keywords, titles and abstracts to enrich the past, present and future analysis by establishing a delimitation from 2000 to 2022, which marked step 1 for the bibliometric study.

The bibliometric structure that shows networks in the research constituents contributes to the intellectual structure founded upon clusters of pertinent themes in the disability and climate change field. Step 2 was executed by triangulating co-citation analysis, bibliometric corpus and co-word analysis techniques for the present study. Step 3 comprised the data collection for the bibliometric analysis explained as augmented by the Preferred Reporting Items for Systematic Reviews and Meta-Analyses (PRIMSA)-Scoping Review illustrated in Figure 1.

### Data collection

The co-authors of the present study have complementary competencies in that the first author has disability expertise, and the latter is a climate change expert. The authors brainstormed terms in this study and identified disab* and climate change as the primary search terms. In addition, the Scopus database was selected as the data source because: (1) it indexes research conducted in the Global North and South. (2) It is one of the largest international databases containing comprehensive research on climate change and disability fields (Sánchez et al., 2017). It is, therefore, relevant to utilise Scopus as a data source.

The formulation of research questions established step 1 in data generation. The research questions include the following:

- Which co-authorship and co-words (keywords, written content or words) exist within climate change and disability as joint fields of study in the Scopus database?
- Which are the most influential publications on the sub-population groups among persons with disabilities covered within disability and climate change research in Scopus?
- Which foundational, present, or periodic themes (socio-economic issues) flagged in the UNCRPD of 2006, and SDGs of 2015 are current in academic debates?
- Which existing and/or future relationships among topics, angles or dimensions were raised and documented from the disability and climate change lens as a joint field of study?

Disab* and climate and change were the primary keywords established for an electronic search in the database Scopus only (Step 2). It, therefore, implies that the search in the Scopus database involved screening using articles (literature), titles, abstracts and keywords. While searching, literature identification and screening ensure the quality of the review using the criteria presented in Table 1 (Step 3). In Step 3, as of July 2023, searching for research work in English within Scopus identified 581 documents.

**TABLE 1:** Inclusion and exclusion in data extraction from the Scopus database.

Guiding aspects	Exclusion criteria	Inclusion criteria
Search field	-	Title, Abstract, Keywords
Keywords:	Impairments	‘Disab*’ and ‘Climate’ and ‘Change’
Open access	Restricted access	All
Years	Before 2000	2000–2023
Author name	Anonymous	All authors
Subject field	-	All
Subject area	-	All
Publication stage	Grey literature	Published on open access
Document Type	Non-peer-reviewed documents and reports.	Articles, reviews, conference papers, conference reviews, books, book chapters, notes, editorials, letters, surveys, data papers and peer-reviewed reports
Affiliation	-	Authors affiliations
Funding sponsor	-	Project funders
Country	-	Origin of the study and the study sample
Source type	Non-academic sources	Academic journals, books, book series, conference proceedings and trade journals
Language	Non-English	English and full-text

The time span for the study was from 2000 to 2022 (more than two decades). It was considered the delimitation period for the search to have sufficient research on climate change and disability as a joint field. The first electronic search produced 581 results. After removing 190 documents by filtering using the inclusion and exclusion criteria in Table 1, the study selected 391 documents only. After reading 391 abstracts, 120 were found to be duplicated documents. To ensure that precise, comprehensive knowledge about climate change and disability is understood, documents that mentioned search terms, while not closely linked to the research purpose, were excluded. These amounted to 120 articles with no available full abstracts, 56 had no full texts on open access, while 99 did not focus on disability and climate change in a single study.

**FIGURE 2:**
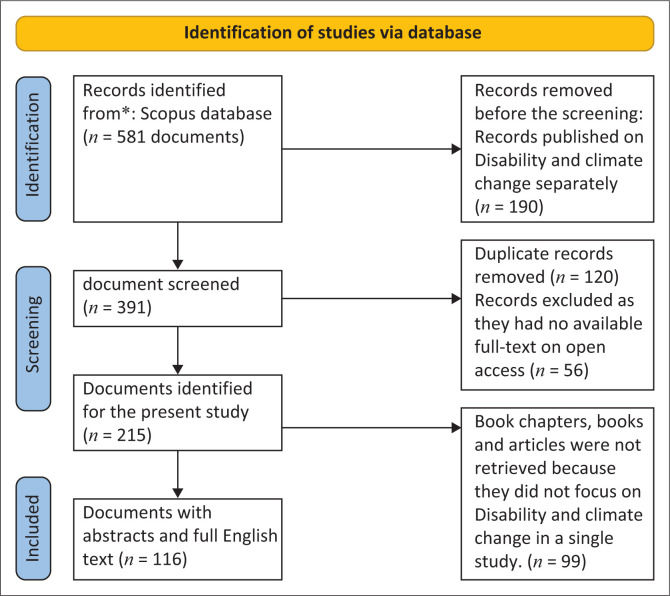
Illustration of the steps in identifying and screening data gathered from Scopus.

Only 116 documents had available abstracts. Reading the 116 abstracts ensures their appropriateness to the selection criteria. Another search in Scopus was conducted by looking through the reference lists of selected documents to provide essential contributions. After the screening process, 116 papers were eligible for literature analysis. Based on PRIMSA for Scoping Reviews (ScR) suggested by Tricco et al. (2018), which is an extension of the work of Page et al. (2020), a flow chart in Figure 2 lists the selection criteria, the number of documents screened, included and excluded.

**Adopted in parts from Page et al. (2020) and Tricco et al. (2018):** Step 4: Running the bibliometric analysis and reporting of the findings followed the electronic search in Scopus generated data from 116 documents, single studies on disability and climate change. The data were exported in CSV file format for bibliometric analysis, using two bibliometric indicators: (1) co-authorship and (2) co-words within climate change and disability as joint fields of study in the Scopus database. These can provide insights into the most influential publications on the sub-population groups among persons with disabilities covered within disability and climate change research in Scopus. This can enable the study to establish foundational, present or periodic themes (socio-economic issues) flagged in the UNCRPD of 2006, and SDGs of 2015 are present in academic debates. Such a review would uncover existing and/or future relationships among topics, angles or dimensions raised and documented from the disability and climate change lens as a joint field of study.

The researchers uploaded the file in CSV format into the VOSviewer software version 1.6.5 (Step 4). VOSviewer is an effective research tool for the enrichment toolbox (Donthu et al. 2021) and nurture validation for systematic literature review because it can construct and view bibliometric maps and identify term clusters and their reference network summaries (Cavalcante, Coelho & Bairrada 2021). The researchers present the findings in the following section.

### Ethical considerations

Ethical clearance to conduct this study was obtained from the Independent Institute of Education, IIE Ethics Committee (reference: R.00092 [REC]).

## Results

### The state of research on Disability and climate change in Scopus

The findings reflect a clear understanding of the coverage and changes documented by research indexed in Scopus on disability and climate change for the past 22 years; the researchers reviewed documents put into two periods, namely pre- and during the COVID-19 pandemic eras.

**FIGURE 3:**
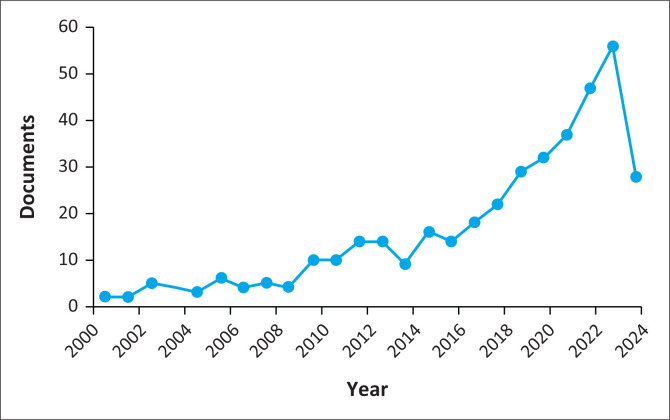
Document per year (2000–2022).

Only 116 documents that focused on disability and climate change are in Scopus. However, based on Figure 3, period 1 (2000–2019), the yield of research publications could be much higher. One scientific publication was there in 2000 and 2001. Slow growth with ups and downs from a minimum of 1 to a maximum of 4 per year between 2000 and 2008, and according to Figure 3, the period spanning 2009–2012 showed improvements in output from 4 to 13 (documents) compared to previous years, followed by a decline in 2013 to 10 documents. The year 2014 showed 16 published documents, and 2015 showed a drop to 13. A positive change in scientific studies on climate change and disability rose from 18 in 2016 to a climax of 56 documents by 2021. In 2022, a sharp decline in the research output reflects 38 publications only. Notably, full-text reading uncovered the fact that early research focused on explaining the vulnerability and existence of discrimination against disability in climate change legislative frameworks (period 1). Within the same period, the narrative slowly evolved to consider socio-economic dimensions and specific population groups, like mental health among people with disabilities, from a health professional lens.

The findings indicated that in period 2 (2020–2022), the health pandemic did not disrupt the growth of research in disability and climate change. This is evident in the increase in publications to 47 in 2020. In 2021, the publications increased to 56, which marked the peak because, in 2022, there was a decline to 38. A full-text reading of the documents uncovered the fact that research conducted between 2020 and 2022 incorporated the self-representation of persons with disabilities, as encouraged by UNCRPD, to complement the narrative with experts’ perspectives (professionals) only. In addition, in period 2, research in the Global South emerged with contexts that share some differences in disability and climate change debates in the Global North. Such views incorporate challenges faced by persons with disabilities, and solutions like involving organisations for people with disabilities can enable the co-creation of measures to alleviate challenges such as extreme weather events leading to flooding. However, a common aspect in Global North and South is disability, and climate change has not yet covered all spectrums of disabilities and sub-groups within the population of people with disabilities.

#### Prominent journals that published research on disability and climate change

**FIGURE 4:**
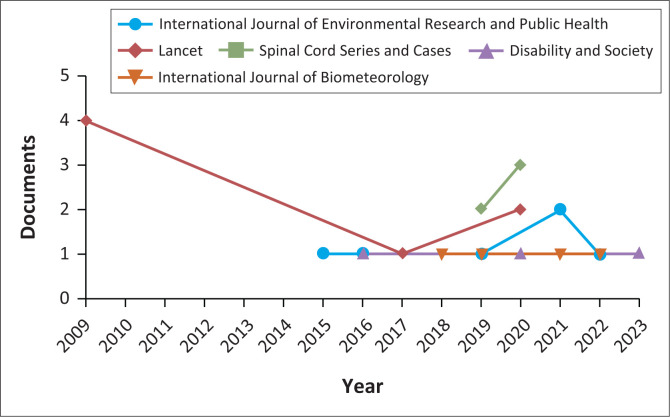
Documents per year by source (top 5 journals on disability and climate change).

The study uncovered the fact that sources of documents found in Scopus on disability and climate change began in 2009, only in the *Lancet* (see Figure 4). Generally, the findings in Figure 4 reflect the top five journals indexed in Scopus with scientific research on climate change and disability as a joint field of study. One can say that a nexus of disability and climate change as phenomena nurtured novelty in broadening the view that everyone is affected in various ways and scales. Research interests grew over time among academic journals, especially after 2014 (see Figure 4).

In addition, the findings reflected in Figure 4 present only the top five journals indexed in Scopus using document count in relationship to the years set as the delimitation of the study. Among the top five journals, the *Lancet* published more on climate change and disability in 2009 with four articles, and there was a break until 2017, when it had one article. In 2020, the Lancet had two articles since 2017. Therefore, from 2000 to 2022, the *Lancet* had seven documents on climate change and disability.

**TABLE 2:** Ten top-ranked countries with researchers on climate change and Disability.

Continents and countries investigated	Position	No. of co-authors of scientific documents
United States of America	1	134
United Kingdom	2	66
Australia	3	47
South America and/or Canada	4	30
**Asia:** China	5	23
**Europe:**		
New Zealand	6	15
Italy	7	14
Asia: India	8	12
**Europe:**		
Switzerland	9	12
Germany	10	11

No., number.

Furthermore, Figure 4 reflects the fact that the *International Journal of Environmental Research and Public Health* followed the *Lancet* in 2015 by publishing a consistent number of single documents per year from 2015 until 2019. It issued no work on disability and climate change in 2020 and published two documents in 2021. In 2022, it returned to its usual single document status. According to Figure 4, the *International Journal of Environmental Research and Public Health* has the same number of documents (seven) as the *Lancet*.

Figure 4 indicates that the spinal cord series and cases began to publish two scientific research studies on disability and climate change as a joint field in 2019. It then published three in 2020, making it have five research outputs as of 2022. Hence, it is ranked third among journals indexed in Scopus that have published scientific work on disability and climate change from 2000 to 2022. In addition, the *International Journal of Biometeorology* published one document each year, which is in 2018, 2019, 2021 and 2022 (see Figure 4), leading it to have four documents on disability and climate change as a single field of study. It then was ranked fourth among journals indexed in Scopus, which have published on disability and climate change as single studies. In addition, Figure 4 reflects the fact that *Disability and Society* began to publish research on Disability and climate change in 2016. It maintained its consistent annual amount of one document in 2016, 2018 and 2020, respectively. It established a total of three documents as of 2022. Such an overall scientific output positions *Disability and Society* as the fifth journal to publish on disability and climate change as a joint field of study indexed in Scopus.

### Co-authorship on Disability and climate change in Scopus

Within the VOSviewer analysis, co-authorship analysis focused on quantifying the frequency with which a specific publication or author publishes (Seguí-Amortegui et al., 2019). It can provide a means to evaluate the research contribution to intellectual structure within a research constituent of a specific field (Cavalcante et al. 2021). In this context, it is on disability and climate change: (1) co-authorship that identifies lead authors and (2) co-word reflects their contribution from health, social, business and management science dimensions within the field of study. With this in mind, the findings reflect 122 authors who co-authored research on climate change and disability in a single study.

Of the 122 authors, nine top-ranked co-authors with more than one document on climate change and disability in Scopus are as follows: Marcalee Alexander has authored the highest number of records, namely 12. Of the 12, six are co-authored with other scholars; nonetheless, all 12 research outputs focused on climate change and people with mental health conditions from a psychiatric professional lens – Marcalee Alexander, affiliated with the University of Alabama School of Medicine in the United States of America. Zaid Chalabi, affiliated with the University College London, United Kingdom, has four. In contrast, Andy Haines, affiliated with the London School of Hygiene and Tropical Medicine in the United Kingdom, has three documents they authored. On the one hand, Ben. G. Armstrong from the World Health Organization, Switzerland, co-authored with other researchers to establish two studies on disability and climate change. Fitzpatrick, Christopher, Mathieu Bangert and Dirk Engels (co-authors) are the latter team of researchers from the World Health Organization, Switzerland, and authored two documents. Alison C. Rivett, Timothy G. Harrison, Dudley E. Shallcross and Anwah H. Khan affiliated with the University of Bristol, United Kingdom, and Jauyah Tuah with the Ministry of Education, Bandar Seri Begawan, Brunei Darussalam), and Gregor Wolbring affiliated with the University of Calgary, Canada among others have single studies on disability and climate change as co-authors.

The findings reflect that the rest of the co-authors (one hundred and three authors) have authored a single document each, respectively. Interestingly, there are tendencies for co-authorship within the climate change and disability research community. The extant literature reflects the narrative spearheaded by health practitioners, and OPDs who later joined the debate towards the end of period 1. Few social scientists and business and management researchers joined the academic discussion in period 2. In the latter period, some authors, like Alexander (2020b), embedded SDGs and UNCRPD perspectives in their research with a legislative-skewed narrative for disability and climate change. Others, like Stein and Stein (2020;2021), analysed the climate change agreements like the one made in Paris in period 2. However, in periods 1 and 2, there is no contextualised climate change definition of disability for a better interpretation of disability-inclusive climate change measures.

### Countries of co-authors

The findings uncover the fact that eight of the 103 focused on Global South countries like China, India and Brunei Darussalam. In addition, within Global South, African countries like Zimbabwe and Malawi were dealt with by researchers who affiliate with an international organisation whose operations are in selected countries. At the same time, the rest of the co-authors considered Global North, which has countries in the United Kingdom like Britain, the United States of America, Europe, Switzerland, and South American countries like Canada. As Nzo et al. (2023) indicated, the findings reflect the geospatial distribution of disability and climate change research as uneven in the Global North and South. As Nzo et al. (2023) ascertained, the extent of disability inclusion into other life-related phenomena like economic empowerment and climate change is determined and influenced by the level of socio-economic and legislative inclusion and ‘disability politics’ in the Global North and South. In addition, ignorance, attitudes, and communication barriers are prevalent among climate change experts on disability and disability inclusion and general societal inclusivity within the states and research communities (Makuyana & Nzo, 2020)

#### Countries and/or territories covered in disability and climate change

The results of VOSviewer show the connection between documents found in Scopus on disability and climate change in a single study and the top 10 countries where the authors focused their research on climate change and disability. Generally, in period 1, the Global North (the United States of America, the United Kingdom and Australia, to mention a few) is leading in research on climate change and disability. This can be attributed to their advancement in universal designs to cater to the ageing population, which shares similar needs with persons with disabilities, and enforceable disability acts (Makuyana & Nzo, 2020)

Findings indicate that while the end of period 1 reflects the fact that the Global South is lagging, countries like Bangladesh, Burkina Faso, Madagascar, Nigeria, Sierra Leone and Zimbabwe were considered in one study as territories and/or spheres of influence for MPHSS (Lunga et al., 2019). In period 2, Mexico and Doom Island were considered in this climate change and disability discourse by researchers, including Ampnir, Santoso and Maturbongs (2022) and González and Alvarado (2019), respectively (see Figure 4). Figure 4 indicates China and India in the top 10. At the same time, Botswana (Dintwa, Letamo & Navaneetham 2019), Brazil (Menezes et al., 2019) and Bangladesh (Arman et al., 2019; Ahmed et al. 2019) are part of the climate change and disability-related debates that emerged in period 1. The rest of the scientific documented knowledge in Scopus focused on the Global North. In period 2, Ahmed et al. (2021) investigated the effect of extreme weather events on injury, disability, and death in Bangladesh. Arman, Salam Shaoli and Hossain (2022) looked into mental health and climate change in the same country. Thus, Morris et al. (2018) looked at disability and climate change from an extreme weather event within the political determinants for persons with disabilities after assessing disaster risk in the aftermath of Hurricane Maria in Puerto Rico-Brazil.

The findings indicate that Simpson et al. (2021) collaborated with persons with disabilities to study disaster and emergency preparedness among people with disabilities in Mexico. Simpson et al. (2021) uncover the unmet needs of persons with disabilities and input on addressing these issues through grassroot coalition building and inclusion. The findings reflect the same urgency as seen in the 2018 IPCC Report; however, Simpson et al.’s (2021) assertion conveys the perspective of those who have lived through disastrous events in Mexico. Simpson et al. (2021) indicated the need to promote emergency and disaster preparedness among people with disabilities, achievable through an intersectional, inclusive, equity-based approach to human rights.

The results from VOSviewer, presented in Table 1, indicate the ten top-ranked countries and/or territories with researchers investigating climate change and disability in single studies between 2000 and 2022.

The rest of the co-authors’ countries of focus (where research emerged from) for their research on climate change and disability between 2000 and 2022 had less than ten, and the least had one document per country. Therefore, geographical distribution is related to how policy-makers and decision-makers in organisations and academic research communities in various geo-political spaces perceive disability. The situation even goes to the extent of legislative support and funding or sponsorship of research on disability and climate change issues. Global South, especially Africa, has a handful of research because of ignorance of disability inclusion, limited accountability and enforceability of legislative tools that address disability issues (Chataika et al. 2019).

#### Co-word analysis results on Disability and climate change in Scopus

It is important to note that the first data extraction was conducted from Scopus and had an analysis of the results (obtained documents). The study focused on the connection between documents by year, year and source, author and country and/or territory. The researchers conducted the second data generation and co-occurrence analysis using data extracted from Scopus in CSV format using VOSviewer. The second VOSviewer enables the establishment of co-occurrences of keywords. The co-occurrence analysis allows for understanding the strengths of links between keywords from the content. The analysis simultaneously evaluates the ranking of the frequency of usage of the keywords in extracted documents (Cavalcante et al. 2021). The linkage strength in the network visualisation of the VOSviewer shows the size of the bubbles. The thickness of the lines between them reflects a higher frequency of co-occurrence. Network visualisation demonstrates the strength of the association between items (Cavalcante et al. 2021). Therefore, the distance between the bubbles in network visualisation reflects the number of documents with the items used in the electronic search on the database (Seguí-Amortegui et al., 2019), in this case, Scopus.

**FIGURE 5:**
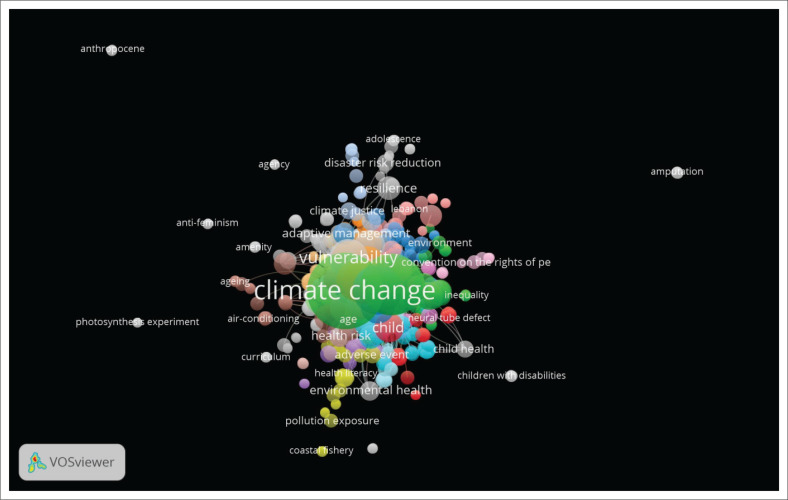
Mapping of co-occurrence of keywords using network visualisation.

The VOSviewer results indicate climate change has 60 occurrences and 452 total link strength. In contrast, disability has 25 occurrences and 176 links. A disabled person has 12 occurrences and 115 total link strength; disabled persons have nine occurrences and 97 total link strength. Aged has eight occurrences and 108 total link strength. In comparison, mental health has nine occurrences and 82 total link strengths. The network of all keyword occurrences and link strengths is shown in Figure 5.

The VOSviewer results indicate climate change as having a giant bubble; however, it links to all keywords. Climate change is a global challenge that diversely affects persons with various disabilities. From the present review, a consensus is that climate change induces and exacerbates vulnerability among people with disabilities. Thus, climate change-induced effects like extreme weather events such as floods threaten people with mobility challenges because of, for instance, spinal cord injuries when intending to escape the floods (Alexander 2019). On the one hand, humans are second and third largest.

The findings indicate that, on the one hand, disability and vulnerability are ranked fourth and fifth. This is because all literature on climate change suggests the vulnerability of persons with disabilities like the ones with spinal cord injuries as they have challenges in mobility (Alexander 2020a, 2020b, 2020c) in the Global North. On the other hand, the vulnerability of mental health conditions in the Global South relates to the effects of climate change, like droughts and cyclones (Lunga et al., 2019) (see also Figure 5). Results of VOSviewer’s clustered thematic words.

**FIGURE 6:**
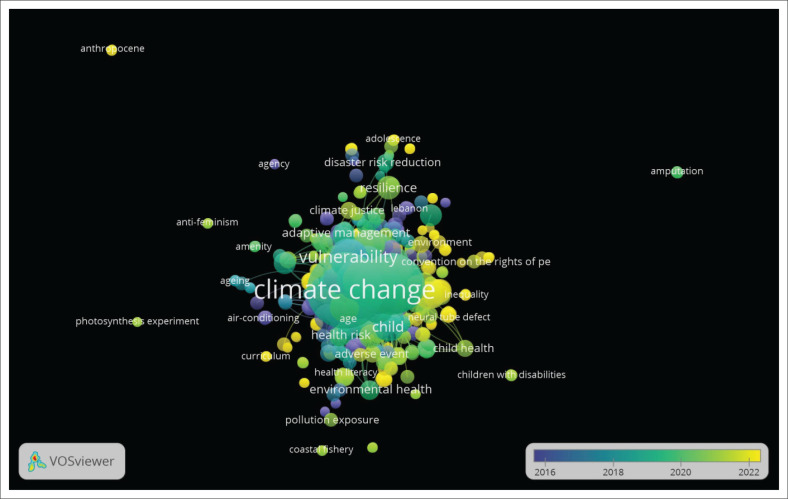
Mapping keywords’ link strength.

Results from VOSviewer indicate that the size of the string indicated in green shows interdisciplinary, intradisciplinary, and multi-disciplinary research in the period 2018–2020 related to disability, climate change and vulnerability (see Figure 6). Figure 6 presents the 802 links, with 2276 total link strength, of climate change to other keywords like disab* (aged included) research as gaining the research community’s interest. The study in yellow indicates terms like adaptive management, climate justice and convention for the rights of persons with disabilities that emerged in research in post-2020, which is skewed to mental health (see Figure 6).

### Themes and suggestions for Disability and climate change research in the future

The findings uncovered that, as opposed to period 1, period 2 shows Alexander (2019, 2020a) as bringing other layers of concerns about the safety of people with spinal cord injuries amidst extreme weather events induced by climate change like floods and heath pandemics like the COVID-19 pandemic. Alexander’s (2019, 2020a) concerns share similarities with other aetiology of disability as part of processes to learning experiences of persons with disabilities for future climate change-induced events.

The findings uphold Maisel et al. (2021) as bringing to the surface the need to bring climate change through inclusive design within the adaptive capacity lens. Within the same line of thought, Engelman Craig and Iles (2022) present case studies from different global regions to illustrate how disability is absent when planning climate-related health impacts and disaster responses. Engelman et al. (2022b) reveal the role of aid from networks led by people with disabilities in adapting to climate-related health impacts. In addition, suggested (raised questions) areas to help policy-makers and practitioners integrate disability justice into their work. Engelman et al. (2022a) opine that the inclusion of people with disabilities, their organisations, and service providers in the co-ownership and co-creation process of developing policies and actions to prevent, enhance, prepare for and respond to climate disasters.

The findings reveal that researchers like Costello et al. (2009), Mörchen et al. (2021), and Lasky et al. (2023) produced letters published to express concern if climate change, vulnerability, disability, climate justice and ableism are not perpetuating inequalities (leaving others behind). The trend indicates a call to build an inclusive climate movement by including disability in climate change debates and/or discussions (Butler et al. 2022). Furthermore, King et al. (2021) propose a teaching and learning guide for disability and climate justice.

#### Disability subgroups covered within climate change research in Scopus

The findings reveal that, towards the end of period 1, Alexander et al. (2019) explored climate change and disability from an angle of the educational needs of rehabilitation professionals in line with disaster management and spinal cord injuries. The line of discussion is more about clinical and/or health practitioners presenting an argument for their patients and/or clients. Furthermore, in period 2, Alexander (2021) considered the nexus of climate change and health from a concern about how people with health conditions like multiple sclerosis, spinal cord injury, visual loss and autism are dealt with by professionals in the climate crisis. One can say that the ones left out of climate change research, including deaf people, are vulnerable to the negative impacts of climate change-induced events. In addition, there is a need to be explicit on how the quality of life of persons with disabilities can be part of the intervention for climate change-induced extreme events (Alexander 2021).

The findings indicate that Sullivan et al. (2022) focused on climate change-induced health effects evident in the health of people with disabilities because there are research gaps in the physiatric literature that must use education. Taylor et al. (2022) concur that there is a need for the physiatric literature to fill the research gap on climate change. Sullivan et al. (2022) suggested the whole medical education continuum to prepare physiatrists to address the climate-related health effects impacting their patient. In addition, physiatrists and their member organisations should actively advocate for policies that address climate change and the needs of their patients while including people with disabilities in the policy-making process, as supported by the UNCRPD of 2006.

Furthermore, Eaton et al. (2022) discussed the need to bring mental health and well-being within climate change to inform future practices (intentions). Eaton et al. (2022) took insights from a Global South lens of mental health and psychosocial support (MHPSS) activities linked to climate change-related emergencies in Bangladesh, Burkina Faso, Madagascar, Nigeria, Sierra Leone and Zimbabwe. In addition, people with intellectual disabilities and climate change have not been explicitly considered in academic fora (Long et al., 2014). Therefore, Lunga et al. (2019) unravel the need for a relook to bring persons with disabilities into the civil protection decision-making and policy formulation in Zimbabwe, an example of a developing country lagging in climate change and disability.

#### Incorporation of SDGs and UNCRPD in climate change academic debates

The researchers familiarised themselves with data by reading each document that was part of the results from an electronic search in Scopus. The researchers deduced emerging themes from the reviewed literature as guided issues raised by authors, dimensions of the discourse, subgroups among the persons with disabilities, alignment to UNCRPD of 2006 and SDGs of 2015, and countries and/or territories covered within disability and climate change. Such analysis enabled the exploration of the latent meaning in the academic debate on Disability and climate change.

The findings from full-text reading uncovered a contrasting pattern. In period 1, the guiding principles were absent in the SDGs and UNCRPD within climate change and disability debates. In period 2, a narrative change was observed compared to the previous period because researchers like Alexander (2020b) incorporated the SDGs within the climate change debate as a layer and dimension to enable health equity for persons with disabilities caused by spinal cord injury. Furthermore, Alexander (2020b) indicated the need to increase the sharing of international knowledge of the challenges of people with spinal cord injury and sicknesses associated with spinal cord injury in developing countries to make necessities available within climate change adaptive and mitigating mechanisms from an informed point of view.

Furthermore, Eriksen et al. (2021) explained reasons and justifications for the importance of understanding the rights of persons with disabilities in the development and rollout of climate-resilience pathways from a UNCRPD lens. Bell et al. (2020) indicated SGDs’ explicit interest in addressing equality and poverty among people with disabilities; however, they propose to obtain insights from disability studies for climate change migration discourse, policy, and processes and practices to incorporate systemic inclusion.

The findings present that, while emphasising limitations in climate change policy’s inclusion of persons with disabilities (Salkeld, 2016) across the globe, disability rights in international governance on climate change have been acknowledged for international legislative instruments for climate change (Jodoin et al. 2021). Despite this acknowledgement, there is still a need at individual-country and local-community levels to have disability-inclusive climate change mitigation and adaptive measures that incorporate all subgroups in the disability space. It implies that climate change and disability research (academic), non-academic narrative, and community-based interventions and initiatives should cater to all subgroups like deaf, visually impaired and blind, to mention a few. Such a broader coverage is essential to reasonably accommodate the approximately 15% who have a form of disability (Makuyana & Nzo, 2022).

This study uncovered the fact that countries in the Global South had given little attention to climate change and disability from health and non-health practitioners, policy-makers and scholars compared with those in the Global North. Countries develop measures to combat climate change and adapt to its impacts without understanding how their initiatives can be designed and implemented in ways that nurture respect, protect and fulfil the human rights of people with disabilities (Jodoin et al. 2021). Therefore, Jodoin introduced a human rights model of disability that embeds UNCRPD for climate governance (Jodoin et al. 2021). The model identifies the differential impacts of climate change for persons with disabilities while establishing the principles, obligations and standards critical in developing and using accessible climate mitigation and adaptation policies and programmes (Jodoin et al. 2021). This can enable a more inclusive world with disability-inclusive climate solutions that can have resonant outcomes to help the disability sub-population groups contribute to carbon neutrality initiatives to enhance the climate resilience of society (Jodoin et al. 2021).

The findings uncovered the fact that while acknowledging the inclusion of disability in international frameworks on climate change, Stein and Stein (2022a; 2022b) considered the Paris Agreement as overlooking persons with disabilities in climate change mitigation and adaptation efforts as a violation of agreed-upon human rights obligations. Their proposition points to a need to incorporate participatory justice that empowers persons with disabilities for climate resilience approaches that are efficacious for, successfully implementable by, and accountable to people with disabilities. King and Gregg (2022) share similar views on understanding how physical disabilities relate to perceptions of climate-related risk and adaptations to climate-related events. King and Gregg (2022) propose a critical realist model of climate justice that aims to comprehend the nexus between environmental features like disabling factors, risk perception, information seeking, adaptive capacity and resilience to climate change. Understanding disabled people’s vulnerability and capacity to adapt to climate change will enable a better understanding of how vulnerable populations cope with climate change and mainstreaming them into climate action and policy.

It, therefore, has implications for a need to have joint knowledge co-creation among international frameworks to protect the rights of persons with disabilities, OPDs, and international organisations that are forming ‘climate-resilient development pathways’ (Oxfam, n.d.; GWP & UNICEF n.d) – supported by scientific findings from the IPCC (IPCC 2018). Climate resilience is increasingly recognised as being inherently political (Schipper et al., 2020). However, efforts often do not sufficiently engage with context-specific socio-ecological, cultural and political processes, including structural inequalities underlying historically produced vulnerabilities (Grasham et al. 2021), like persons with disabilities and ageing people (Nzo et al., 2023). It implies that politicised approaches pose barriers to concerted and meaningful change as they increase the chances of concealing the agency of different actors and obfuscating roles and responsibilities (Ferguson 2019). Therefore, depoliticising climate change can contribute to alleviating some vulnerable and marginalised population groups among persons with disabilities who are left out. It thus implies that climate change experts should incorporate disability into the climate-resilience concept. This is essential because climate resilience, in essence, cuts across sectors and disciplines and is prone to varying interpretations, resulting in differing and sometimes competing definitions (Moser, 2019). Hence, there is a need to co-create knowledge on climate change that upholds the self-representation of all persons with disabilities while being guided by UNCRPD.

There are implications that climate change policy and decision makers within the resilience strategising space for pathways for the diversified communities may perpetuate the ‘blanks’ because of the absence of knowledge on all people with disabilities. Such a knowledge gap may adversely or negatively affect the interpretation of UNCRPD and SDGs to the effect of a continued exclusion among persons with disabilities in climate change mitigation and adaptive measures. In addition, climate resilience legislative tools aimed to build and enable resilience among all diverse people in communities may further exclude some groups among persons with disabilities. Therefore, the study suggests collaboration between researchers, policy-makers and implementing agencies to engage vulnerable communities continuously. Such engagement can inform and provide insights to facilitate and empower individual decision-making, identification, understanding and communicating risks to all persons with disabilities using communicable means. It, therefore, implies that local contextualised concerns and research evidence-based approaches on disability and climate change can lead to better provision of support and promotion of adaptation in various ways.

## Conclusion

The study concludes after a bibliometric review in Scopus using keywords like disab* and climate change. The co-word analysis found the words in various disciplines ranging from medical to environmental and social sciences perspectives. Co-authorship analysis on disability and climate change as joint fields of study in the Scopus established the fact that the Global North has more co-authors who contributed to the extant research intelligence compared to the Global South. It, therefore, implies uneven coverage of knowledge on disability and climate change in fields such as health, environment, business and/or management and socio-economics, both in the Global North and South. Global South is lagging, which implies that the geospatial distribution of publications relates to the geo-political-based influence of disability movement. However, compared to the Global South, the Global North has been leading in pushing the inclusion agenda by generating knowledge to inform climate change legislative tools.

Interestingly, the mental health (psychiatric) and other psychosocial sub-groups of persons with disabilities received research attention above the rest within disability and climate change research in Scopus. It, therefore, implies a need to broaden the scope and balance knowledge co-creation with children, youth, women and ageing among other people with disabilities sub-groups like blind and partially blind people, deaf and hard of hearing people, people of short stature, people with autism, mobility-impaired people and multiple disabilities.

Based on the findings, the conclusion is that extant literature on disability and climate change indexed in Scopus has not yet uncovered the linkage of disability classification within the climate change context. It, therefore, has implications that policy-makers and policy-implementing agents in the climate change space need to establish disability classification from a climate change lens. It can enable easier adoption of disability definitions within climate change guidelines and pathways. Therefore, the study indicates themes and gaps emerging from extant literature for future research. Consequently, this poses real-life implications for societies’ inclusive climate resilience beyond natural and built environments, attitudes, and communication for effective climate resilience pathways that do not perpetuate ‘othering’ norms. Therefore, the authors recommend the multi-disciplinary lens research on disability and climate-change to reduce compounded vulnerabilities and inequities experienced by hard of hearing, deaf, visually impaired people, blind, mobility-impaired people, speech-impaired, and those with multiple disabilities who are not in the literature found in Scopus.

In conclusion, therefore, it poses implications for the definition of disability adopted by the present study. It lacks climate change context, and the interpretation is subjective to one’s perspective. That is, disability is the complex interaction between the ways that disability is constructed (individual and social constructivism), experienced, the interplay between individual interpretation and shared understanding, the private versus social self, and the politics of impairment versus socio-environmental disablement (Olsen & Pilson, 2022). The definition of disability has implications for the climate resilience discourse aimed at furthering knowledge co-creation and application as climate resilience pathways that include everyone in the global village. Climate-change resilient development is the emerging paradigm within international sustainable development in theory and practice (Grasham et al. 2021). So, with limited climate-disability research in the Global South, persons with disabilities can continue to be marginalised and left out in climate resilience debates and real-life practice.

Therefore, the research community in the climate change space should incorporate the UNCRPD lens within the climate change discourse. In addition, governments of countries in the Global North and South should foster disability-inclusive policies for climate-resilient economies driven by international frameworks. However, the review reflected an absence of Disability in international frameworks like the Paris Agreement (Grasham et al. 2021). At the same time, researchers from various countries have different paces in the knowledge generation lens. In contrast, others have made efforts beyond policy formulation.

The study provides insights for a future research agenda that can foster the co-creation of disability and climate change knowledge that can help decision-makers, researchers and vulnerable communities. It, therefore, implies multi-stakeholder and transdisciplinary approaches towards collaborative work that link and deliberate research-based programmes designed to enable resilience to climate change-related hazards and risks to every person with disabilities. On the one hand, the study had limitations in only considering one database, Scopus. On the other hand, it recommends further studies using other databases while augmenting with empirical studies for all persons with disabilities.
